# A Six-Week Smartphone-Based Program for HPV Prevention Among Mothers of School-Aged Boys: A Quasi-Experimental Study in South Korea

**DOI:** 10.3390/healthcare12232460

**Published:** 2024-12-05

**Authors:** Yun-Hee Cho, Tae-Im Kim

**Affiliations:** 1 Department of Nursing, Jeonbuk Science College, Jeongeup 56204, Republic of Korea; cyh7151@jbsc.ac.kr; 2 Department of Nursing, Daejeon University, Daejeon 34520, Republic of Korea

**Keywords:** health education, human papillomavirus, human papillomavirus vaccine, male, mothers

## Abstract

Background: Human papilloma virus (HPV) affects both males and females, but in South Korea, vaccination rates for boys are significantly lower due to cultural stigma and limited awareness. Effective strategies are needed to close this gap. Methods: This study evaluated a 6-week smartphone-based HPV prevention program for mothers of school-aged boys, designed using the extended theory of planned behavior (E-TPB). The program aimed to enhance knowledge, attitudes, subjective norms, and self-efficacy, with the goal of increasing vaccination intention and uptake. The E-TPB incorporated knowledge as a key element to improve behavioral intention and vaccination uptake. A nonequivalent control group pre-test–post-test design included 54 mothers (28 in the experimental group and 26 in the control group). Results: The experimental group showed significant improvements in HPV knowledge (*p* < 0.001; d = 1.41), HPV vaccine knowledge (*p* < 0.001; d = 1.13), attitudes (*p* < 0.001; r = 0.48), subjective norms (*p* = 0.014; d = 0.61), self-efficacy (*p* < 0.001; r = 0.53), and vaccination intention (*p* < 0.001; r = 0.58). The experimental group achieved a vaccination uptake rate of 25.0%, compared to 4.0% in the control group, representing a six-fold increase (RR = 6.25; *p* = 0.033; h = 0.64). Conclusions: The program effectively addressed key factors influencing vaccination behavior, leading to significant increases in HPV vaccination rates among boys. Smartphone-based education shows promise in reducing gender disparities in vaccination uptake, though further studies with larger samples are needed to validate these findings.

## 1. Introduction

Human papillomavirus (HPV) is a prevalent sexually transmitted infection that affects both sexes and is associated with several types of cancer, including cervical, anal, oropharyngeal, and penile cancers [1]. In the U.S., HPV-associated cancers impact 657,317 individuals, with women accounting for 59.8% and men 40.2% of these cases [2]. These statistics highlight the significant burden HPV imposes on both sexes. Globally, there is an increasing trend in non-cervical HPV-associated cancers, particularly oropharyngeal cancer, which occurs more frequently in men. In Korea, the incidence of oropharyngeal cancer is notably higher among men [3].

HPV vaccination is most effective when administered to boys and girls aged 9–14, prior to their sexual debut, as it can prevent HPV-associated cancers. Vaccination is cost-effective, providing immunity with two doses when administered before the age of 15. When given to both boys and girls, it can induce herd immunity, thereby maximizing the preventive effect against HPV [1]. Considering that the average age for the first sexual experience among Korean adolescents is 13.1 years [4], there is a pressing need for active strategies to boost HPV vaccination rates among boys aged 11–12.

Despite these trends, HPV prevention efforts have predominantly focused on females, creating a gender gap in vaccination programs and public awareness [5,6]. In South Korea, cultural stigma and the perception of HPV vaccination as a “female-only” vaccine hinder vaccination efforts for boys, who are often excluded from national immunization programs [7,8]. These societal misconceptions and inadequate public health messaging fail to emphasize the protective role of vaccination for males against non-cervical cancers and HPV transmission.

In South Korea, the HPV vaccination rate for boys is alarmingly low, ranging from 0.7 to 1.3% [9,10,11], compared to 35.0–43.8% for girls [12]. Additionally, studies suggest that maternal education on HPV plays a crucial role in the decision-making process regarding the HPV vaccination for young children [8,11,13,14]. However, HPV awareness and intent to vaccinate among mothers of school-aged boys remain significantly low due to the absence of educational programs targeting boys and their families [9,10,11]. Addressing this gap requires interventions that enhance maternal knowledge, challenge cultural barriers, and promote gender-neutral vaccination strategies. This study aims to address these challenges by developing and evaluating a smartphone-based educational program for mothers of school-aged boys, guided by the extended theory of planned behavior (E-TPB).

The extended theory of planned behavior (E-TPB) was selected for this study due to its comprehensive framework and predictive power for health-related behaviors, especially vaccination uptake. The E-TPB extends the traditional TPB by incorporating subjective norms and additional modifiable variables such as knowledge, which are critical for addressing maternal decisions around HPV vaccination [15,16,17,18,19]. Unlike the Health Belief Model (HBM), which focuses on perceived susceptibility, severity, benefits, and barriers, the E-TPB explicitly addresses external social pressures through subjective norms, making it highly relevant in collectivist cultures like South Korea [5]. Subjective norms play a significant role in maternal decision-making, as research highlights the significant role of societal and familial expectations in shaping vaccination behaviors [5,20]. Additionally, the E-TPB’s flexibility to include knowledge as a construct aligns with evidence showing that educational interventions can enhance vaccination rates by addressing awareness gaps [19,21,22]. By targeting both internal motivations (attitudes, perceived control) and external influences (social approval), the E-TPB provides a robust framework for this intervention.

With the rise in remote learning post-COVID-19, smartphone-based education has become a popular and effective method for engaging parents, offering self-directed, accessible learning opportunities that overcome the limitations of time and space [23,24].

Therefore, this study was designed to develop and assess a smartphone-based education program on the prevention of HPV infection (EPP-HPVI) for mothers of school-aged boys. The program was designed to improve HPV-related knowledge, attitudes, self-efficacy, and vaccination intentions among mothers of school-aged boys, ultimately addressing the low HPV vaccination rates among boys in South Korea. This intervention was grounded in the extended theory of planned behavior (E-TPB) to enhance its theoretical robustness.

## 2. Materials and Methods

### 2.1. Research Design

This study used a nonequivalent control group pre-test–post-test design to investigate the effectiveness of the smartphone-based EPP-HPVI.

### 2.2. Participants

The study participants were mothers of male students in the fourth to sixth grade from two elementary schools in a South Korean city. The schools were selected for their comparable socioeconomic status (average monthly household income, education level) and student–teacher ratios [25,26], ensuring similar demographics and minimizing the potential for selection bias. The 20 km distance between the schools helped prevent the contamination of experimental effects, with mothers assigned to the experimental or control group based on the school their child attended.

The sample size was determined using the G*Power 3.1.9.4 program. Based on a previous study [21], with a significance level of α = 0.05, a power of 1-β = 0.80, and an effect size of d = 0.20, the estimated sample size was 42 (21 in each group). To accommodate for potential dropouts, 56 mothers were recruited (28 in each group). Two participants in the control group dropped out prior to the intervention due to work-related travel. As these dropouts occurred before the intervention phase, no systematic bias is expected. The final sample consisted of 26 participants in the control group and 28 in the experimental group.

**Figure 1:**
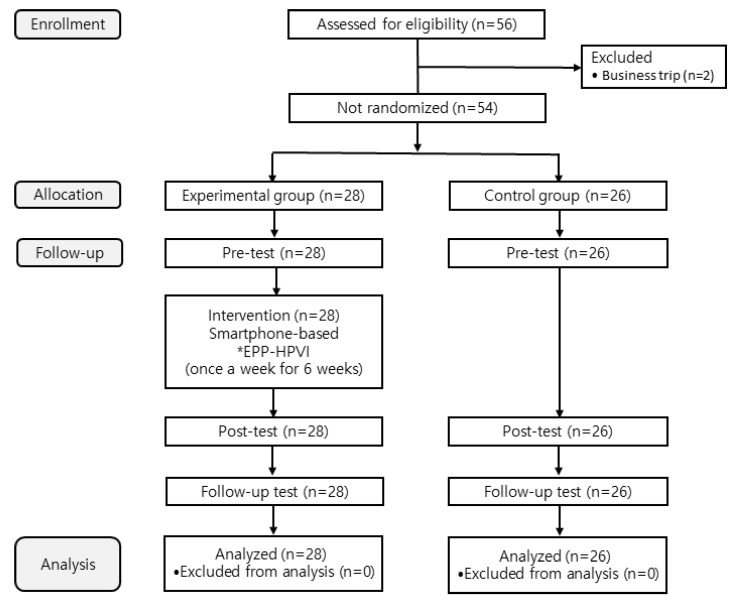
Data collection procedure flow diagram. GC = General characteristics; HRC = HPV-related characteristics; HK = HPV knowledge; HVK = HPV vaccine knowledge; ASV = Attitude toward sons’ vaccination; SNV = Subjective norms related to HPV vaccination; SEV = Self-efficacy related to HPV vaccination; HVI = HPV vaccination intention; HUR = HPV vaccine uptake rates; EPP-HPVI = Education program for the prevention of human papilloma virus infection. ^(1)^ Immediately after the intervention, ^(2)^ 8 weeks after the intervention.

The inclusion criteria were (1) the son had not been vaccinated against HPV, (2) the mother had not participated in any HPV educational programs in the past six months, (3) the mother understood the purpose of the study and provided written consent, and (4) the mother was capable of using a smartphone. The participant selection process, including the number of participants excluded and the final sample size, is detailed in Figure 1.

### 2.3. Measures

A structured questionnaire was employed to explore sociodemographic and HPV-related characteristics, along with E-TPB constructs. Permission was obtained from the original developers to use all instruments.

#### 2.3.1. Sociodemographic and HPV-Related Characteristics

The sociodemographic and HPV-related characteristics included age, education, occupation, monthly household income, the son’s age, the awareness of HPV and the HPV vaccine, the mothers’ own HPV vaccination status, and their intention to vaccinate their sons against HPV if the vaccine were covered by health insurance.

#### 2.3.2. HPV Knowledge

The HPV knowledge tool, developed by Waller (2013) [27], revised by Sherman (2018) [28], and translated into Korean by Kang (2019) [9], was used. This tool consists of 23 true–false items, with possible scores ranging from 0 to 23. The scale demonstrated a Kuder–Richardson 20 (KR-20) value of 0.89 in Kang’s study and 0.84 in this study.

#### 2.3.3. HPV Vaccine Knowledge

The HPV vaccine knowledge tool, developed by Waller (2013) [27], revised by Sherman (2018) [28], and translated into Korean by Kang (2019) [9], was used. This tool consists of nine true–false items, with possible scores ranging from 0 to 9. The scale demonstrated a Kuder–Richardson 20 (KR-20) value of 0.68 in Kang’s study and 0.72 in this study.

#### 2.3.4. Attitudes Toward Sons’ HPV Vaccination

A tool developed by Gerend and Shepherd (2012) [29] and translated into Korean by Kim (2018) [30] was used to measure mothers’ attitudes toward their sons’ HPV vaccination. This 4-item scale uses a 7-point Likert scale, with total scores ranging from 4 to 28. Higher scores indicate more positive attitudes. Cronbach’s α was 0.88 in Gerend and Shepherd’s study, 0.93 in Kim’s study, and 0.92 in this study.

#### 2.3.5. Subjective Norms Related to Sons’ HPV Vaccination

A tool developed by Gerend and Shepherd (2012) [29] and translated into Korean by Kim (2018) [30] was used to measure mothers’ subjective norms related to their sons’ HPV vaccination. This scale consists of six items rated on a 5-point Likert scale, with total scores ranging from 6 to 30. Higher scores reflect stronger subjective norms. Cronbach’s α was 0.78 in Gerend and Shepherd’s study, 0.92 in Kim’s study, and 0.90 in this study.

#### 2.3.6. Self-Efficacy Related to a Son’s HPV Vaccination

A tool developed by Gerend and Shepherd (2012) [29] and translated into Korean by Kim (2018) [30] was used to measure mothers’ self-efficacy related to their sons’ HPV vaccination. This scale consists of six items rated on a 7-point Likert scale, with total scores ranging from 6 to 42. Higher scores indicate greater self-efficacy regarding their sons’ HPV vaccination. The Cronbach’s α values were 0.85 in Gerend and Shepherd’s study, 0.97 in Kim’s study, and 0.89 in this study.

#### 2.3.7. Intention to Vaccinate Sons Against HPV

A tool developed by Gerend and Shepherd (2012) [29] and translated into Korean by Kim (2018) [30] was used to measure mothers’ intention to vaccinate their sons against HPV. This 5-item scale uses a 7-point Likert scale, with total scores ranging from 5 to 35. Higher scores signify a greater intention to vaccinate. Cronbach’s α was 0.96 in Gerend and Shepherd’s study, 0.96 in Kim’s study, and 0.90 in this study.

#### 2.3.8. HPV Vaccination Rate

The HPV vaccination rate was assessed 8 weeks after the intervention. A coding scheme was employed where “0” indicated individuals who did not receive the HPV vaccine, and “1” indicated those who received one or more doses.

### 2.4. Program Development

The EPP-HPVI was developed based on the E-TPB [15,29] and followed the procedures outlined in the analysis, design, development, implementation, and evaluation (ADDIE) model [31].

In the analysis phase, to construct the themes and content of the EPP-HPVI, a literature review was conducted on factors influencing maternal HPV vaccination for their children and on intervention studies. Additionally, to understand the educational needs of the participants, focus group interviews were conducted with four mothers of elementary school-aged boys.

In the design and development phase, we created the EPP-HPVI based on the E-TPB. The EPP-HPVI was designed to enhance knowledge, attitudes, subjective norms, and self-efficacy regarding HPV and the HPV vaccine, with the goal of increasing mothers’ intention to vaccinate their sons against HPV and, ultimately, raising their sons’ HPV vaccination rates. A smartphone-based program utilizing SNS (KakaoTalk) was developed, which included two instructional videos, five webtoons (see Supplementary Figure S1), a booklet, and two additional media to boost engagement. Public commitment was promoted through slogan chanting [32]. The draft was evaluated by experts, including two women’s health nursing professors and two school health teachers with over 17 years of experience. Revisions were made based on their feedback.

In the implementation and evaluation phase, a pilot application of the revised EPP-HPVI was conducted with four mothers of elementary school-aged boys, with the session times and content adjusted based on their feedback. The final version of the EPP-HPVI, after conducting the preliminary research, is presented in Table 1.

### 2.5. Research Procedure

This study was conducted from May to August 2022 after obtaining approval from the Institutional Review Board (IRB) of D University in D Metropolitan City. The school principal and the supervising teacher were provided with an explanation of the purpose and methods of the study to obtain permission before starting the study. The mothers who consented to participate and met the eligibility criteria were included.

The pre-test was conducted one week before the six-week intervention through after-school meetings at two schools. After providing consent, mothers completed a questionnaire. Following the pre-test, participants were informed about the post-test (at 6 weeks) and a follow-up test (at 14 weeks), both conducted via Naver Office Form. Research assistants helped participants access the KakaoTalk chat room and Naver Office Form, confirming their participation. An educational booklet and the EPP-HPVI program schedule were provided to the experimental group.

The experimental group participated in weekly smartphone-based educational sessions, each lasting 30–100 min, once a week for 6 weeks. Participants in the control group received no treatment during this period. After the intervention, a post-test was conducted using Naver Office Form to assess outcomes. Eight weeks after the intervention, a follow-up test was conducted, using Naver Office Form, to evaluate mothers’ intentions regarding their sons’ HPV vaccination and the actual vaccination rates among their sons. During the eight-week follow-up period, participants in the experimental group received weekly encouragement messages via KakaoTalk and were asked to respond to these messages to monitor their adherence.

**Figure 2:**
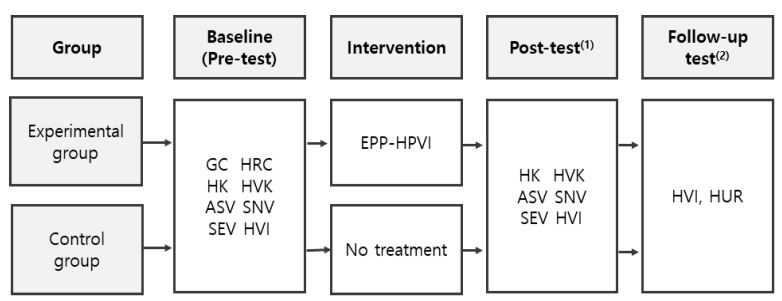
Flowchart of participant selection and analysis. *EPP-HPVI = education program for the prevention of human papilloma virus infection.

Attendance was checked before each session, and active engagement during the sessions was encouraged to ensure the consistent delivery of the educational content and maintain intervention fidelity. No HPV-related campaigns or public health initiatives targeting HPV vaccination were conducted in the study area during the intervention and follow-up periods, minimizing the likelihood of external influences on the study results. After completing the follow-up test, participants received online gift vouchers as a reward. Additionally, educational materials were shared with the control group after the study was completed to ensure ethical considerations were met. The data collection procedure is shown in Figure 2.

### 2.6. Data Analysis

The collected data were analyzed using SPSS/WIN 22.0 (IBM Corp., Armonk, NY, USA). Descriptive statistics (frequency, percentage, mean, standard deviation) were used to summarize participants’ characteristics. Data normality was assessed with the Shapiro–Wilk test, and the homogeneity of variances was tested with Levene’s test. Normally distributed variables were analyzed using the independent *t*-test, while non-normally distributed variables were analyzed using the Mann–Whitney U test. The group homogeneity was evaluated using the χ^2^ test, Fisher’s exact test, and *t*-tests.

Intervention effects were evaluated by comparing means (Mann–Whitney U test) and proportions (Fisher’s exact test) between groups. Effect sizes (r, h) and the relative risk (RR) were calculated, and power analysis was conducted using G*Power 3.1.9.4.

### 2.7. Ethical Considerations

This study was approved by the Institutional Review Board (IRB) of D University in D Metropolitan City (IRB No. 1040647-202110-HR-010-03). Before the study commenced, the school principal and the supervising teacher were provided with an explanation of the study’s purpose and methods to obtain their permission. To ensure confidentiality, the questionnaires were sealed immediately after distribution and completion. Participants were informed that they could discontinue completing the questionnaire at any time during the study and that the data would not be used for any purpose other than the study. Written consent to participate was obtained after the participants received an explanation of the study.

## 3. Results

### 3.1. Participant Selection Process

The participant selection process, including the number of participants screened, excluded, and analyzed, is illustrated in Figure 2.

### 3.2. Homogeneity Testing of Sociodemographic and HPV-Related Characteristics and Outcome Variables at Baseline

There were no significant differences in the sociodemographic or HPV-related characteristics between the experimental and control groups. Furthermore, the outcome variables revealed no significant differences between the two groups, confirming their homogeneity (Table 2).

### 3.3. Evaluation of the Effectiveness of the Smartphone-Based EPP-HPVI

#### 3.3.1. After the Intervention

The experimental group showed significant improvements across all outcome measures compared to the control group:

- HPV knowledge: The experimental group improved by 9.11 points, while the control group improved by 1.0 point. This difference was statistically significant (t = 5.83; *p* < 0.001) with a large effect size (Cohen’s d = 1.41).
- HPV vaccine knowledge: The experimental group increased by 2.71 points, compared to 0.35 points in the control group. This difference was statistically significant (t = 3.82; *p* < 0.001) with a large effect size (Cohen’s d = 1.13).
- Attitudes toward vaccinating sons against HPV: The experimental group showed a median improvement of four, compared to zero in the control group. The difference was statistically significant (z = 3.89; *p* < 0.001) with a moderate to large effect size (Pearson’s r = 0.48).
- Subjective norms: The experimental group showed a 3.75 point increase, while the control group showed a 1.04 point increase. This difference was statistically significant (t = 2.53; *p* = 0.014) with a moderate to large effect size (Cohen’s d = 0.61).
- Self-efficacy: The experimental group showed a median improvement of 9.00, compared to 2.04 in the control group. The difference was statistically significant (z = 3.89; *p* < 0.001) with a moderate to large effect size (Pearson’s r = 0.53).
- Vaccination intention: The experimental group showed a median improvement of 7.00, compared to 0.38 in the control group. The difference was statistically significant (z = 4.26; *p* < 0.001) with a moderate to large effect size (r = 0.58) (Table 3).

#### 3.3.2. Eight Weeks Post-Intervention:

- Vaccination intention: The experimental group (M = 44.05; median = 44.8 [40.95–48.65]) scored significantly higher than the control group (M = 36.75; median = 39.9 [31.68–42.00]) (z = 3.35; *p* = 0.001; Pearson’s r = 0.46), with a medium-to-large effect size and a statistical power of 0.96. This result highlights the substantial practical impact of the intervention on vaccination intentions.
- HPV vaccine uptake rate: The experimental group achieved a significantly higher vaccine uptake rate (25.0%) compared to the control group (4.0%), representing a sixfold increase in the uptake (RR = 6.25; *p* = 0.033). This indicates that participants in the experimental group were 6.25 times more likely to receive the HPV vaccine than those in the control group. This result showed a moderate-to-large effect size (Cohen’s h = 0.64) with a statistical power of 0.71, likely limited by the small sample size (Table 4).

Overall, the intervention significantly improved HPV knowledge, vaccine knowledge, attitudes, subjective norms, self-efficacy, and vaccination intentions, with effect sizes ranging from moderate to large (Cohen’s d = 0.61–1.41; Pearson’s r = 0.48–0.58), indicating substantial improvements in the experimental group. Furthermore, the substantial increase in vaccination uptake highlights the practical impact of the intervention despite some outcomes being constrained by limited statistical power.

## 4. Discussion

The smartphone-based education program (EPP-HPVI) developed for mothers of elementary school boys significantly improved HPV knowledge, vaccine knowledge, attitudes, subjective norms, self-efficacy, and the intention to vaccinate their sons. It also led to a notable increase in the HPV vaccine uptake rate among school-aged boys.

After the 6-week intervention, the experimental group showed a significant improvement in their HPV and vaccine knowledge compared to the control group. Although direct comparisons are challenging due to variations in participants’ demographics and measurement tools, previous studies have reported that parents’ HPV knowledge increased by a factor of 1.06 to 1.36 times the baseline score post-intervention [33,34,35,36,37]. In contrast, our study recorded a 1.84 times increase, which can be attributed to the intervention’s unique approach. Unlike one-time educational methods, such as face-to-face lectures [33] or video education [34], our study used a 6-week smartphone-based program with periodic uploads of videos and webtoons, enhancing engagement and learning outcomes. Active two-way communication, including discussions and Q&A sessions, enhanced participant engagement and learning outcomes. Furthermore, the comprehensive and evidence-based content of the program also covered important topics, such as HPV and HPV infection, HPV-related diseases and their links to cancers in both genders, the importance of preventing HPV infection and timely HPV vaccination, and the vaccine’s effectiveness, safety, side effects, and adverse reactions, significantly boosting participants’ awareness of the need for vaccination [8,14].

The experimental group also demonstrated a significant increase in attitudes and subjective norms regarding HPV vaccination for their sons, which aligns with previous studies [33,34]. Several factors contributed to these results. First, two television videos were chosen to underscore the social issues associated with HPV vaccination in males, thereby enhancing participants’ awareness. Furthermore, the webtoons featured dialogs among mothers that addressed the link between HPV infection and cancer, employing characters and imagery to subtly promote social awareness about HPV prevention [38].

In this study, self-efficacy regarding HPV vaccination increased significantly in the experimental group. Self-efficacy, which is closely associated with perceived behavioral control within the framework of the TPB, plays a crucial role in predicting preventive health behaviors [18,29]. Our intervention boosted self-efficacy through various techniques, including verbal persuasion, vicarious experience, mastery experience, and emotional arousal [16]. Verbal persuasion was enhanced by lecture videos that provided up-to-date information on HPV and the HPV vaccine; these were also adapted into webtoons to facilitate repeated learning. The webtoons enabled mothers to observe vicarious achievements and grasp the significance of HPV vaccination through dialogs about HPV-related cancer risks and prevention strategies. Emotional arousal was achieved by chanting slogans and sharing emotions [32]. These approaches successfully increased mothers’ self-efficacy regarding their sons’ HPV vaccination.

The experimental group also showed a significant increase in vaccination intention, which lasted up to 8 weeks post-intervention. This result is attributed to the use of the E-TPB framework used in the development of the intervention, which proved effective in enhancing vaccination intention and uptake. These results affirm the effectiveness of the E-TPB framework in predicting and enhancing HPV vaccination intentions and uptake, thereby supporting its practical application [9,10]. Previous studies found that vaccination intention typically lasts 2 to 4 weeks [33,39], but our study extended this effect. This prolonged impact is likely due to the use of more diversified educational content and methods, which proved more effective than traditional lecture-based education [34,37]. Previous research has shown that repeated learning through periodic education significantly boosts HPV vaccination intention and sustains its effect over time. Weekly interventions were found to be more effective than one-time lectures [37]. Although this study did not measure the frequency of access to smartphone content post-intervention, future research should investigate how the frequency of access influences HPV vaccination intention.

Despite these positive outcomes observed in the intervention group, the actual HPV vaccine uptake rate (25%) was lower than expected. Several factors likely contributed to this discrepancy. First, the study was conducted during the off-peak vaccination period in South Korea (May to August), when vaccination rates are typically lower. The winter break (December to February) is a more common time for vaccinations [40], suggesting that seasonal peaks significantly influence vaccination behavior [41]. Many mothers in the experimental group planned to vaccinate their sons during this period, highlighting the impact of timing on vaccine uptake. Future studies should aim to align data collection with peak vaccination periods for more accurate assessments of intervention effectiveness. Second, social stigma around HPV vaccination for boys may also have played a significant role. HPV vaccination is often perceived as a “female-only” vaccine, and its association with sexual health can make it a sensitive topic for parents to discuss with their sons [5]. Many mothers in this study reported difficulties in addressing sexual health topics, reflecting broader cultural barriers in South Korea, where such communication is considered taboo [5]. These findings align with previous research [5,37,42,43] and underscore the need for and importance of school-based education and healthcare provider recommendations to normalize HPV vaccination for boys [5,44]. Studies indicate that Korean mothers highly value school-based education and are notably influenced by healthcare providers, reflecting a cultural tendency to respect hierarchical authority and the esteemed position of doctors in society [5]. In this context, healthcare provider recommendations could be particularly effective in overcoming cultural barriers and addressing parental hesitancy. As trusted sources of health information, providers can clearly communicate the importance of HPV vaccination as a preventive measure against serious diseases, emphasizing its relevance for both genders. Research demonstrates that strong provider endorsements significantly boost vaccination rates, even in stigmatized contexts [5,44]. To address these challenges, future interventions should combine healthcare provider recommendations with parental education to normalize HPV vaccination for boys and dispel persistent misconceptions. Furthermore, efforts should focus on reducing stigmatization and promoting open conversations about HPV prevention. Third, financial constraints also likely contributed to the low uptake rate, as boys are excluded from the national vaccination program in South Korea, making the vaccine a financial burden for many families [45,46]. Despite increased vaccination intentions, these financial and logistical constraints prevented some families from following through with vaccination. Finally, external factors, such as competing health campaigns or fluctuations in public awareness, may also have influenced vaccination decisions during the study period [47]. Future research should be conducted on influences interacting with educational interventions to better understand their collective impact.

Mothers in this study also voiced concerns about effectively conveying the importance of HPV vaccination to their sons. Research suggests that engaging multiple stakeholders—such as school officials, healthcare providers, and adolescents themselves—can enhance vaccination rates [8,14,44,47]. Interventions that involve both parents and children, particularly through school- and community-based programs and healthcare provider recommendations, have proven effective in increasing both the intention to vaccinate and the actual vaccine uptake [5,8,41]. Such programs not only distribute information equitably but also enhance the credibility of the intervention [11,14]. While statistical tests showed no significant baseline differences between the experimental and control groups, some characteristics, such as maternal education and prior HPV knowledge, may still have influenced the outcomes. Although these factors were not statistically significant, they could be relevant in interpreting the intervention’s effects. Future studies should further explore these potential influences on vaccination behaviors.

This study has several limitations. First, the sample was limited to mothers of school-aged boys from two schools in a South Korean city, which may limit the generalizability of the results. Second, the study primarily focused on HPV vaccination intentions and uptake rates, but did not assess the long-term effects on knowledge, attitudes, subjective norms, and self-efficacy. Future research should examine these sustained impacts to provide a more comprehensive understanding of intervention outcomes. Third, while the HPV knowledge scale used in this study demonstrated an acceptable KR-20 reliability coefficient (0.72), consistent with previous studies, the moderate internal consistency may limit its precision, particularly in assessing complex constructs. Future research could employ alternative validated tools with higher reliability to improve measurement accuracy. Fourth, the relatively low statistical power for vaccine uptake (71%) may limit the ability to detect smaller effect sizes. Despite this limitation, the intervention demonstrated a relative risk (RR) of 6.5 (95% CI: 0.74–57.17), suggesting a meaningful increase in vaccination uptake. Additionally, the number needed to treat (NNT) was calculated as five (95% CI: three–twenty-nine), highlighting the practical significance of the intervention. Expanding sample sizes or extending the intervention period could address this limitation and enhance the robustness and generalizability of future studies. Fifth, the use of self-reported data introduces potential recall bias and social desirability bias, which may have influenced the accuracy of responses. Incorporating objective measures, such as vaccination records or data from healthcare providers, is recommended for future studies to validate self-reported outcomes. Lastly, the lack of blinding in the study design may have led to biases in outcome measurements. Future research should consider blinded or randomized controlled designs to minimize these biases.

Despite its limitations, the smartphone-based EPP-HPVI effectively demonstrated the theoretical adequacy of the E-TPB framework by significantly increasing HPV vaccination intentions among mothers of school-aged boys and improving the vaccination rates of their sons. By addressing HPV prevention as a gender-neutral issue, this study broadened the scope of HPV vaccination research to include boys, filling a critical gap in the literature. Although this study targeted mothers of boys, the educational content, tailored for both genders, enhances the program’s applicability for broader populations.

The development of tailored educational materials, including lecture videos, webtoons, and a booklet, made the intervention highly accessible, engaging, and satisfactory, distinguishing it from existing resources and highlighting its potential for scalable public health applications.

## 5. Conclusions

This study utilized the E-TPB framework to develop and evaluate the effectiveness of the smartphone-based EPP-HPVI intervention among mothers of school-aged boys. The intervention significantly improved HPV and HPV vaccine knowledge, attitudes, subjective norms, self-efficacy, the intention to vaccinate sons against HPV, and HPV vaccination rates. These findings underscore the potential of smartphone-based educational programs to address HPV vaccination disparities, particularly for boys, who are often excluded from national vaccination programs.

While promising, the results are limited by the small, specific sample and cannot be generalized to broader populations. Future research should extend the follow-up period to evaluate the sustained effects of the intervention on knowledge, attitudes, self-efficacy, and vaccination behaviors, providing a more comprehensive understanding of the program’s long-term effectiveness. Additionally, replication studies across diverse settings and populations are needed to validate the findings and enhance generalizability. To further understand barriers to vaccine uptake, future studies should adopt qualitative or mixed-method approaches to identify cultural, social, and logistical challenges that may hinder vaccination efforts.

## Figures and Tables

**Table 1:** The content of the smartphone-based education program for the prevention of HPV infection among mothers of elementary school boys.

Theme (Time)	Contents and Activities	E-TPB Strategy
Session 1 Understanding HPV and HPV infection (60 min)	Introduction. Show webtoon (1) and instructional video (1). - HPV and HPV infection: causes, risk factors, signs and symptoms, treatment, complications, and prevention. - HPV-associated diseases and cancers in both sexes. - Men and HPV infection. Show webtoon (1) again. Sharing opinions and feelings about today’s learning topic, engaging in Q&A, and chanting slogans! Closing the session and providing guidance for the next one.	Knowledge Attitude Subjective norms Self-efficacy
Session 2 Prevention of HPV infection and HPV vaccines (60 min)	Introducing today’s class activities and chanting slogans! Show Gardasil advertisement video and a TV drama scene about male HPV vaccination. Show instructional video (2). - Prevention of HPV infection. - ACIP recommendations for HPV vaccination. - HPV vaccines: who needs it, how it works, types, vaccine schedule, uptake rate in both sexes, reasons to get HPV vaccine, HPV vaccine safety, effectiveness, and possible side effects. - HPV vaccine and national immunization program in Korea. Sharing opinions and feelings about today’s learning topic, engaging in Q&A, and chanting slogans! Closing the session and providing guidance for the next one.	Knowledge Attitude Subjective norms Self-efficacy
Session 3 Characteristics of HPV and HPV infection (30 min)	Introducing today’s class activities and chanting slogans! Show webtoon (2). - Men, women, and HPV infection. - Route of HPV transmission. - HPV types and associated diseases. - Early detection of HPV infection in men. Sharing opinions and feelings about today’s learning topic, engaging in Q&A, and chanting slogans! Closing the session and providing guidance for the next one.	Knowledge Attitude Subjective norms Self-efficacy
Session 4 HPV vaccine and prevention of HPV infection (30 min)	Introducing today’s class activities and chanting slogans! Show webtoon (3). - Prevention of HPV infection. - HPV vaccine, vaccination schedule, and procedure. - HPV vaccination in both sexes. Sharing opinions and feelings about today’s learning topic, engaging in Q&A, and chanting slogans! Closing the session and providing guidance for the next one.	Knowledge Attitude Subjective norms Self-efficacy
Session 5 HPV vaccine safety and effectiveness (30 min)	Introducing today’s class activities and chanting slogans! Show webtoon (4). - The reasons why the HPV vaccine should be administered. - HPV vaccine uptake rate in both sexes. - HPV vaccine safety, effectiveness, and possible side effects. Sharing opinions and feelings about today’s learning topic, engaging in Q&A, and chanting slogans! Closing the session and providing guidance for the next one.	Knowledge Attitude Subjective norms Self-efficacy
Session 6 Korea’s national immunization program and HPV vaccination for boys (30 min)	Introducing today’s class activities and chanting slogans! Show webtoon (5). - HPV vaccine and national immunization program in Korea. - Why should the HPV vaccine be administered at the recommended age? Sharing opinions and feelings about today’s learning topic, engaging in Q&A, and chanting slogans! Closing remarks.	Knowledge Attitude Subjective norms Self-efficacy

HPV = human papilloma virus; E-TPB = extended theory of planned behavior; ACIP = advisory committee on immunization practice.

**Table 2:** Homogeneity tests for general and HPV-related characteristics and dependent variables.

Characteristics	Categories	Exp. (n = 28)	Cont. (n = 26)	x^2^ or t or z	*p*
		n(%) or M ± SD or Median [IQR]	n(%) or M ± SD or Median [IQR]		
Mother’s age (years)	30–39	2(7.1)	5(19.2)		0.260 ^(2)^
	40–49	16(57.2)	16(61.6)		
	≥50	10(35.7)	5(19.2)		
		44.25 ± 2.58	43.35 ± 3.07	t = 1.18	0.246
Mother’s education	University	21(75.0)	13(50.0)	x^2^ = 3.61	0.052
	≥Graduate school	7(25.0)	13(50.0)		
Mother’s employment status	Employed	23(82.1)	20(76.9)	x^2^ = 0.23	0.445
	Unemployed	5(17.9)	6(23.1)		
Monthly income of the family (10,000 KRW)	<400	6(21.4)	6(23.1)		0.913 ^(2)^
	400–499	2(7.2)	3(11.5)		
	≥500	20(71.4)	17(65.4)		
Son’s age (years)		12.36 ± 0.62	12.12 ± 0.77	t = 1.28	0.246
Awareness of HPV	Yes	21(75.0)	22(84.6)	x^2^ = 0.77	0.297
	No	7(25.0)	4(15.4)		
Awareness of HPV vaccine	Yes	21(75.0)	21(80.8)	x^2^ = 0.26	0.429
	No	7(25.0)	5(19.2)		
Mother’s HPV vaccination status	Yes	8(28.6)	5(19.2)	x^2^ = 0.64	0.316
	No	20(71.4)	21(80.8)		
Intention to vaccinate their son against HPV when covered by health insurance	Yes	25(89.3)	20(76.9)	x^2^ = 1.48	0.286
	No	3(10.7)	6(23.1)		
Reasons for not vaccinating my son against HPV ^(1)^	Do not know about HPV vaccine	6(50.0)	8(57.1)		
	Boys do not need to get the HPV vaccine	4(33.3)	3(21.4)		
	Concerns about the side effects	1(8.3)	3(21.4)		
	Economic burden	1(8.3)	0(0.0)		
Knowledge of HPV		10.82 ± 6.21	13.73 ± 6.41	t = 1.69	0.096
Knowledge of HPV vaccine		5.29 ± 2.52	5.96 ± 2.55	t = 0.98	0.333
Attitude toward sons’ HPV vaccination		20.00 [20.00–24.00]	24.00 [18.75–24.00]	z = 0.64 ^(3)^	0.522
Subjective norms related to sons’ HPV vaccination		20.46 ± 3.49	19.96 ± 5.28	t = 0.42	0.679
Self-efficacy related to sons’ HPV vaccination		30.00 [24.00–32.02]	30.00 [24.00–36.00]	z = 0.20 ^(3)^	0.840
Intention to vaccinate son against HPV		25.50 [22.25–29.00]	26.50 [23.00–30.00]	z = 0.64 ^(3)^	0.520

HPV = human papilloma virus; Exp. = experimental group; Cont. = control group; M = mean; SD = standard deviation; IQR = interquartile range; KRW = Korean Won. ^(1)^ Exclude mothers who plan to vaccinate their son for HPV, ^(2)^ Fisher’s exact test, ^(3)^ Mann–Whitney U test.

**Table 3:** Effectiveness of smartphone-based HPV infection prevention program for mothers of elementary school boys.

Variables	Categories	Exp. (n = 28)	Cont. (n = 26)	t or z	*p*	Effect Size
		M ± SD or Median [IQR]	M ± SD or Median [IQR]			
Knowledge of HPV	Pre-test	10.82 ± 6.21	13.73 ± 6.41			
	Post-test	19.93 ± 3.18	14.73 ± 6.59			
	Difference	9.11 ± 6.41	1.00 ± 3.14	t = 5.83	<0.001	d = 1.41
Knowledge of HPV vaccine	Pre-test	5.29 ± 2.52	5.96 ± 2.55			
	Post-test	8.00 ± 1.36	6.31 ± 2.54			
	Difference	2.71 ± 2.69	0.35 ± 1.72	t = 3.82	<0.001	d = 1.13
Attitude toward sons’ HPV vaccination	Pre-test	20.00 [20.00–24.00]	24.00 [18.75–24.00]			
	Post-test	26.00 [24.00–28.00]	22.50 [18.00–28.00]			
	Difference	4.00 [1.25–8.00]	0.00 [−1.00–4.00]	z = 3.49 *	<0.001	r = 0.48
Subjective norms related to sons’ HPV vaccination	Pre-test	20.46 ± 3.49	19.96 ± 5.28			
	Post-test	24.21 ± 3.64	21.00 ± 4.03			
	Difference	3.75 ± 3.63	1.04 ± 4.23	t = 2.53	0.014	d = 0.61
Self-efficacy related to sons’ HPV vaccination	Pre-test	30.00 [24.00–32.02]	30.00 [24.00–36.00]			
	Post-test	37.98 [36.00–42.00]	36.00 [23.51–40.52]			
	Difference	9.00 [6.00–12.00]	2.04 [0.00–6.00]	z = 3.89 *	<0.001	r = 0.53
Intention to vaccinate son against HPV	Pre-test	25.50 [22.25–29.00]	26.50 [23.00–30.00]			
	Post-test	32.50 [30.00–35.00]	28.13 [22.50–35.00]			
	Difference	7.00 [5.00–9.38]	0.38 [−1.00–5.00]	z = 4.26 *	<0.001	r = 0.58

Exp. = experimental group; Cont. = control group; HPV = human papilloma virus; IQR = interquartile range; * Mann–Whitney U test. *Note: Attitude, self-efficacy, and intention scores did not follow a normal distribution (p < 0.05, Shapiro–Wilk test) and are presented as the median [IQR].*

**Table 4:** Comparison of vaccination intentions and sons’ vaccination rates eight weeks after the intervention between the two groups.

Variable	Exp. (n = 28)	Cont. (n = 26)	z	*p*	Relative Risk (RR)	Effect Size	Power (1 − β)
	Median [IQR] or N (%)	Median [IQR] or N (%)					
Intention to vaccinate son against HPV	44.8 [40.95–48.65]	39.9 [31.68–42.00]	3.35	0.001	N/A	Pearson’s r = 0.46	0.96
Sons’ HPV vaccine uptake	7 (25.0)	1 (4.0)	-	0.033 *	6.25	Cohen’s h = 0.64	0.71

Exp. = experimental group; Cont. = control group; HPV = human papilloma virus; IQR = interquartile range; * Fisher’s exact test.

## Data Availability

The data that support the findings of this study are available from the corresponding author upon reasonable request.

## Supplementary Materials

The following supporting information can be downloaded at: https://www.mdpi.com/article/10.3390/healthcare12232460/s1, Figure S1: A smartphone screen displaying a webtoon upload interface
