# Supplementary material for: A Six-Week Smartphone-Based Program for HPV Prevention Among Mothers of School-Aged Boys: A Quasi-Experimental Study in South Korea
Source: Healthcare (Basel). 2024 Dec 5;12(23):2460. doi: 10.3390/healthcare12232460 (PMC11641219; doi:10.3390/healthcare12232460)
Supplement: Supplementary file 1 [file healthcare-12-02460-s001.zip › healthcare-3301728-supplementary.pdf]

Supplementary Figures

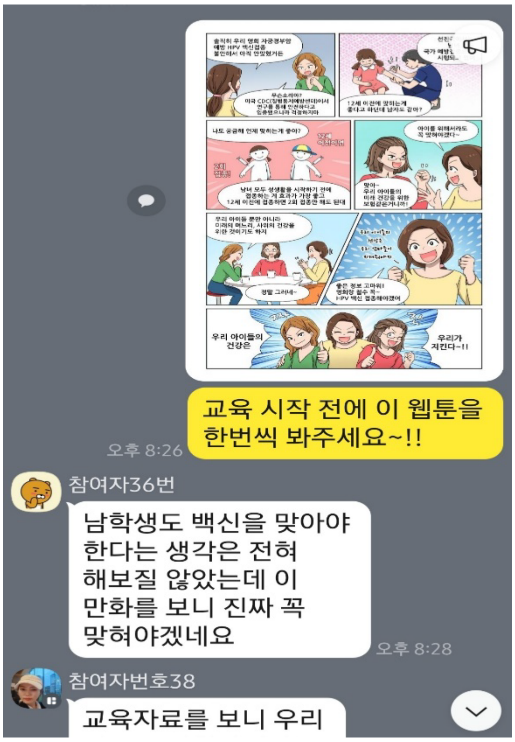

Figure S1. Example of a smartphone screen. (a) A smartphone screen displaying a webtoon upload interface; (b) A smartphone screen sharing the thoughts and feelings of the participants.

■ **The content of the webtoon speech bubbles:**

●Person 1 (woman in yellow): "I haven't vaccinated my child yet because I'm anxious about the HPV vaccine!"

●Person 2 (woman in green): "What are you talking about? The CDC in the US has proven through research that it's safe, so don't worry!"

●Person 1: "I heard it's better to get vaccinated before the age of 12. Is it the same for boys?", "I heard that in developed countries, boys also benefit from the national vaccination program."

●Person 2: "I'm curious too. When is the best time to get vaccinated?"

●Person 3 (woman in red): "It's most effective to get vaccinated before starting sexual activity, and if done before 12, only two doses are needed."

●Person 1: "I definitely need to get my child vaccinated for their sake."

●Person 3: "Yes, it's like insurance for our children's future health."

●Person 2: "Not just for our kids, but also for the health of future daughters-in-law and sons-in-law."

●Person 1: "That's true. Our children's health must be taken care of by their mothers!"

"Thank you for the good information. I'll make sure to get my children vaccinated against HPV."

●They all shout together, "We will protect our children's health!"

■ **Conversation between educator and participant on smartphone screen**

●Educator: "Please take a look at this webtoon before we begin the training."

- Participant 36: "I never thought boys should get the vaccine, but after watching this webtoon, I now truly believe it’s something they must get."
